# Formation and Identification of Unresolved Complex Mixtures in Lacustrine Biodegraded Oil from Nanxiang Basin, China

**DOI:** 10.1155/2014/102576

**Published:** 2014-08-10

**Authors:** Pengfei Guo, Sheng He, Shukui Zhu, Derong Chai, Shiyan Yin, Wei Dai, Wanfeng Zhang

**Affiliations:** ^1^Key Laboratory of Tectonics and Petroleum Resources of Ministry of Education, China University of Geosciences, Wuhan 430074, China; ^2^College of Mining Engineering, Hebei United University, Tangshan 063009, China

## Abstract

A comprehensive two-dimensional gas chromatography/time-of-flight mass spectrometry (GC × GC/TOFMS) method has been developed for the formation and identification of unresolved complex mixtures (UCMs) in lacustrine biodegraded oils that with the same source rock, similar maturity, and increasing degradation rank from Nanxiang Basin, China. Normal alkanes, light hydrocarbons, isoprenoids, steranes, and terpanes are degraded gradually from oil B330 to oil G574. The compounds in biodegraded oil (oil G574) have fewer types, the polarity difference of compounds in different types is minor, and the relative content of individual compounds is similar. All the features make the compounds in biodegraded oil coelute in GC analysis and form the raised “baseline hump” named UCMs. By injecting standard materials and analyzing mass spectrums of target compounds, it is shown that cyclic alkanes with one to five rings are the major components of UCMs. Furthermore, UCMs were divided into six classes. Classes I and II, composed of alkyl-cyclohexanes, alkyl-naphthanes, and their isomers, are originated from the enrichment of hydrocarbons resistant to degradation in normal oils. Classes III ~ VI, composed of sesquiterpenoids, tricyclic terpanes, low molecular steranes, diasteranes, norhopanes, and their isomers, are probably from some newly formed compounds during the microbial transformation of oil.

## 1. Introduction

Biodegraded oils exist widely in many oil and gas basins in the world. According to the statistics, 10% of global oil reserves have been degraded and another 10% have suffered varying degrees of biodegradation [1]. Although it is more difficult to be exploited than normal oil, biodegraded oil has still drawn great attentions as an important type of crude oil. The biodegradation of crude oil is essentially an oxidation reaction associated with microbes. The volume and API of biodegraded oil are smaller, while its density, viscosity, and content of nonhydrocarbon gases, resin, and trace elements are bigger than those of normal oil [2–4]. Another feature of biodegraded oils is the “baseline hump” in gas chromatograms. It has been concluded that the “hump” contains thousands of compounds named as unresolved complex mixtures (UCMs) [5]. Ventura et al. (2008) analyzed UCMs in a bitumen sample from late Archean sediments using comprehensive two-dimensional gas chromatography (GC × GC), and the results showed that early- to mid-eluting UCMs were dominated by polycyclic compounds, and late eluting UCMs were composed of C_36_ ~ C_40_-mono-, bi-, and tricyclic isoprenoids [6]. UCMs also exist in the artificially weathered Norwegian Sea crude oil, and Melbye et al. (2009) identified them as cyclic and aromatic sulfoxide compounds and benzothiophenes [7]. Additionally, UCMs in oils with different degradation levels usually have different features. Tran et al. (2010) studied a group of oils with different sources and degradation ranks in order to describe the oil biodegradation and identify the composition of UCMs [8]. It was proposed that alkyl-decahydronaphthalenes constitute a significant contribution to UCMs. Wang et al. (2012) analyzed UCMs in the heavy oils from Liaohe oilfield of China [9]. The results suggested that UCMs can be divided into two parts (C_24_− and C_24_+). The former is composed of monocyclic, bicyclic, and tricyclic alkanes and the latter is mainly composed of tetracyclic and pentacyclic alkanes. However, there are still lots of works that need to be done in order to determine the formation and composition of UCMs in biodegraded oil.

In this study, UCMs in five lacustrine biodegraded oils with different biodegradation ranks from Nanxiang Basin, China, were separated with comprehensive two-dimensional gas chromatography/time-of-flight mass spectrometry (GC × GC/TOFMS) and identified by standard materials validation, structured chromatograms, and mass spectrums of target compounds. The geochemical characteristics, degradation ranks of the biodegraded oils, and formation and identification of UCMs were studied in detail.

## 2. Geological Setting

**Figure 1:**
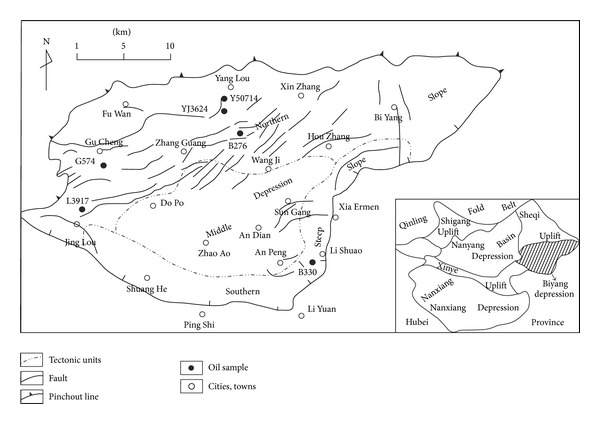
Location, tectonic units, and sampled wells of Biyang depression.

The Mesozoic to Cenozoic Biyang depression is one of the depressions in Nanxiang Basin, which is a typical lacustrine basin located in Henan Province, Eastern China. Biyang depression can be divided into three structural units, the northern slope, the middle depression, and the southern steep slope (Figure 1). The metamorphites in Qinling and Erlangping group of the north Qinling fold belt are the basement of the depression. This basement is overlain by series of sedimentary formations, such as the Eocene Dacangfang formation, the Eocene to Oligocene Hetaoyuan formation (Eh), the Oligocene Liaozhuang formation, and the Miocene Fenghuangzhen formation [10–12]. Hetaoyuan formation was formed in the rift stage of the depression and can be divided into the upper (Eh_1_), the middle (Eh_2_) and the lower (Eh_3_). Eh_3_ in the middle depression was deposited in a deep lacustrine environment and was up to 2000 m thick. Black to grey mudstones were the dominant lithology of Eh_3_, with the high abundance of organic matter and the appropriate maturity [13]. Therefore, the Eh_3_ is the major source rocks of Biyang depression. Eh_3_ in the edge of the depression was deposited in a deltaic sedimentary environment (fan delta and braid river delta), and the dominant lithology was sandstones, a good reservoir of the northern slope [13]. Oils produced from the reservoirs in northern slope are almost biodegraded oils as their depth is lower than 1000 m.

## 3. Experimental Methods

### 3.1. Samples and Chemicals

Five biodegraded oils from the northern slope and one normal oil from the southern steep slope were analyzed (Figure 1). Biodegraded oils' reservoirs are the sandstones in Eh_3_^2^, with the depth ranging from 263.0 m to 1083.0 m. The six oils' group compositions are shown in Table 1. The relative content of resin in biodegraded oils (oils B276 to G574) is higher than that of normal oil (oil B330) obviously.

A suitable amount (35 mg) of oil was dissolved in 30 mL hexane and then mixed for 5 min using ultrasonic wave. The samples need to stand for 12 hours and the asphaltene was filtered. At last, the supernatant was analyzed by GC × GC/TOFMS, GC/MS, and GC, respectively. Standard materials were purchased from J&K Scientific and solvents come from Tedia High Purity Solvent.

### 3.2. Instrumentation and Methods

The GC × GC system consisted of a GC (7890A, Agilent Technologies, Wilmington, DE, USA) equipped with a secondary oven and a quad-jet dual stage modulator. A detailed description of the cold-jet modulator has been made in a previous publication [13, 14]. Two capillary columns were connected serially by means of a Siltek treated universal press-tight connector (Restek Corp., Bellefonte, PA, USA). A time-of-flight mass spectrometer (Pegasus 4D, Leco Corp., St. Joseph, MI, USA) was used to acquire mass spectral data of the effluents from the GC × GC. Data acquisition and processing were performed using a ChromaTOF software version 4.33. The GC/MS system consisted of a 7890A gas chromatograph and a 5975C mass spectrometer (Agilent Technologies, Wilmington, DE, USA). The GC system consisted of a 7890A gas chromatograph equipped with a flame ionization detector (FID) (Agilent Technologies, Wilmington, DE, USA).

In GC × GC/TOFMS analysis, a DB-5MS column (60 m × 0.25 mm × 0.25 *μ*m) and a DB-17Ht column (1.6 m × 0.25 mm × 0.25 *μ*m, J&W Scientific, Folsom, CA, USA) were used as the primary and secondary dimensional columns, respectively. The carrier gas was helium (purity ≥ 99.9995%) with a flow rate of 1.2 mL/min. The injector temperature was 300°C. Injections were performed in the splitless mode, and the injection volume was 1.0 *μ*L. All injections were made with a 7683B series autosampler. The 1st oven temperature was programmed to 60°C (holding for 1 min) and then heated to 310°C at 2°C/min (holding for 30 min). The 2nd oven temperature was 10°C higher than that of the 1st oven. The modulation period and the temperature offset higher than the primary oven temperature of modulator were 6 s and 15°C, respectively. The mass spectrometer was operated at an acquisition rate of 100 spectra per second for a mass range of 50 to 550 u, using electron impact ionization mode at 70 eV and 1500 V multichannel plate voltage. The ion-source temperature was 230°C and the transfer-line temperature was 300°C. The pressure inside the flight tube was 1.1 × 10^−7^ Torr.

In GC/MS analysis, a DB-5MS column (50 m × 0.25 mm × 0.25 *μ*m, J&W Scientific, Folsom, CA, USA) was used. The carrier gas was helium (purity ≥ 99.9995%) with a flow rate of 1.0 mL/min. The injector temperature was 300°C. Injections were performed at the splitless mode, and the injection volume was 1.0 *μ*L. All injections were made with a 7683B series autosampler. The oven temperature was programmed to 60°C (holding for 1 min) and then heated to 300°C at 3°C/min (holding for 30 min).

In GC analysis, a DB-Petro column (30 m × 0.25 mm × 0.25 *μ*m, J&W Scientific, Folsom, CA, USA) was used. The carrier gas was nitrogen (purity ≥ 99.9995%). The flow rate, injector temperature, volume, autosampler, and oven temperature program were the same as the GC/MS analysis.

## 4. Results and Discussion

### 4.1. Geochemical Characteristics

#### 4.1.1. Source Rocks

The Eh_3_ Formation can be divided into the Eh_3_^1^ and the Eh_3_^2^. Literatures have shown that biodegraded oils from the Eh_3_^2^ sandstones in the northern slope came from the Eh_3_^2^ mudstones [14–16]. The biodegraded oils in this study all come from the Eh_3_^2^ sandstones, so their source rocks are the same, that is, the Eh_3_^2^ mudstone.

#### 4.1.2. Maturity

Biomarker maturity indicators, such as sterane maturity index (C_29_ *α* *α* *α*20R/(S + R) and C_29_ *α* *β* *β*/(*α* *α* *α* + *α* *β* *β*)), are usually used to assess the maturity of oil [17–19]. Steranes and terpanes will not be depleted when the oil degradation rank is lower than 5 [19, 20]. Therefore, the sterane maturity index can be applied to calculating the maturity of oils (degradation rank lower than 5) from the northern slope of Biyang depression. The results show that the oils are low mature oils. In addition, the maturity of the Eh_3_^2^ source rocks was discussed by Dong et al., and the results suggested that the Ro of the Eh_3_^2^ is about 0.5% to 0.7% [13]. Through the above analyses we can conclude that biodegraded oils B276 to G574 are low mature oils with similar maturity.

#### 4.1.3. Biomarker Features

The biomarkers of the oils in the northern slope were analyzed by GC/MS. Different parameters of oils (degradation rank lower than 5) are calculated (Table 2). The relative content of C_27_-, C_28_-, and C_29_-regular steranes can indicate the original organic matter inputs of source rocks [20]. The dominance of C_27_-regular sterane indicates the input of aquatic organic matter, C_29_-regular sterane indicates the input of land-plant organic matter, and C_28_-regular sterane can reflect the organic matter from the algas [20]. The oils in the northern slope have a relative high content of C_27_- and C_28_-regular sterane (0.27~0.32 and 0.31~0.34), which suggests the dominant input of lacustrine organic matter. The composition of tricyclic terpanes is an effective organic matter input index [20]. The oils in the northern slope have a low ratio of C_19_/C_23_-tricyclic terpanes or C_19_ ~ C_22_/C_23_ ~ C_26_-tricyclic terpanes and a high ratio of C_26_-tricyclic terpane/C_24_-tetracyclic terpane. It also proves the dominance input of lacustrine organic matter. Furthermore, oleanane that represents land plants input was not detected in the oils. Therefore, the oils in the northern slope are typical lacustrine oils.

In addition, biomarker features can reflect the oil maturity, such as T_s_/(T_s_ + T_m_), C_27_ ~ C_29_-diasteranes/steranes, the content of low molecular weight steranes, tricyclic terpanes, and homohopanes [20]. The ratios of T_s_/(T_s_ + T_m_), C_31_ ~ C_35_-homohopanes/C_30_-hopane, C_21_ ~ C_22_-pregnanes/(steranes + diasteranes), C_19_ ~ C_29_-tricyclic terpanes/*M*/*Z*: 191, C_19_ ~ C_29_-tricyclic terpanes/C_30_-hopane, and C_27_ ~ C_29_-diasteranes/steranes are 0.10~0.15, 0.54~0.61, 0.01~0.03, 0.23~0.32, 0.97~1.49, and 0.05~0.06, respectively. All the ratios are relatively low. This also suggests that the oils in the northern slope are low mature oils.

### 4.2. Degradation Rank

Compounds in crude oil have different kinds of capability resisting the biodegradation, especially the biomarkers [21–24]. Some studies have empirically demonstrated that the resistance to biodegradation of n-alkanes, isoprenoids, steranes, pentacyclic triterpenoids, tricyclic diterpenes, diasteranes, 25-norhopane, 25, 30-bisnorhopane, tetracyclic diterpenes, 22,29,30-trisnorhopane, and gammacerane increases gradually [20]. Oil degradation rank has been divided into 10 ranks by Wenger (2002) according to the composition of biomarkers [19]. In this study, the degradation ranks of six oils were determined based on Wenger's degradation standard.

**Figure 2:**
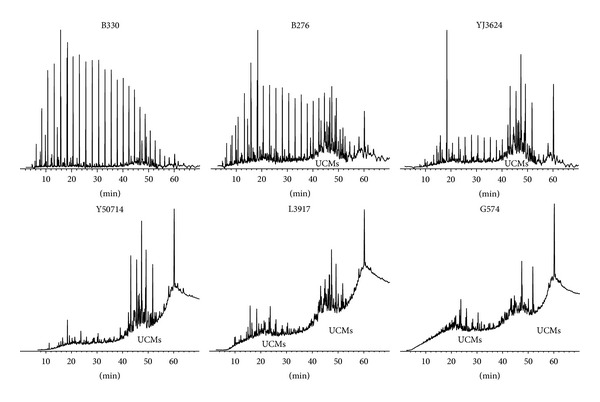
GC chromatograms of oil B330 to oil G574.

The overall composition of the crude oil, such as the composition of n-alkanes and the relative content of steranes and terpanes, can be characterized by GC analysis. The GC chromatographs of six oils are shown in Figure 2. The n-alkanes of oil B330 were hardly degraded, which indicates that oil B330 is normal oil. N-alkanes and isoprenoids of oil B276 to oil G574 were degraded gradually. Steranes and terpanes in oil L3917 began to be degraded and were seriously degraded in oil G574. The “baseline hump” (UCMs) enlarges gradually from oil B330 to oil G574 (Figure 2). These features indicate that the degradation rank of oils B330 to G574 increases gradually, oil B330 is normal oil, and the degradation ranks of oils B276 and YJ3624 are ranks 2 and 3.

In order to determine the degradation ranks of oils Y50714 to G574, the profiles of steranes and terpanes need to be further compared. The degradation rank of oil YJ3624 is rank 3 with steranes and terpanes not being degraded. The relative content of diasteranes, low molecular weight steranes, and tricyclic terpanes in oil YJ3624 is low, and it is the same with that of normal oil B330. Similar composition of oil Y50714 and oil YJ3624 indicates that neither steranes nor terpanes in oil YJ3624 have been degraded. Steranes and terpanes in oil L3917 began to be degraded, and the relative content of diasteranes, low molecular weight steranes, and tricyclic terpanes is higher than that in oil YJ3624 and oil Y50714. Only some steranes and terpanes resistant to degradation, such as low molecular weight steranes, diasteranes, tricyclic terpanes, norhopanes, and gammacerane, were detected in oil G574, and the highest peaks of* M*/*Z*: 217 and* M*/*Z*: 191 are C_21_-pregnane and gammacerane, respectively. The above features indicate that the degradation ranks of oil Y50714 to oil G574 are 5, 6, and 8.

The above analysis shows that oil B330 is normal oil and oil B276 to oil G574 are biodegraded oil with degradation ranks of 2, 3, 5, 6, and 8. Oils B276 to G574 are all low mature oils and their source rock is the same (black mudstones in Eh_3_^2^). So the original compositions of oil B276 to oil G574 are comparable. Therefore, this set of oils can be used for the study on the formation of UCMs in biodegraded oil.

### 4.3. Formation of UCMs

The UCMs (“baseline hump”) can be clearly observed in oil B276 to oil G574. UCMs are a typical complex system, and their compounds may be as many as 25,000 [7]. Due to the limitation of separation capacity, UCMs can be hardly separated by GC and GC/MS [25]. In order to analyze the generation and change of UCMs, GC × GC/TOFMS was used to characterize the geochemical composition of the biodegraded oils [26, 27]. The baseline separation and orthogonal separation of oil G574 were achieved by GC × GC/TOFMS analysis. Then the relative content, types, and polarity differences of compounds in different biodegraded oils were analyzed.

#### 4.3.1. Relative Content of Compound

**Figure 3:**
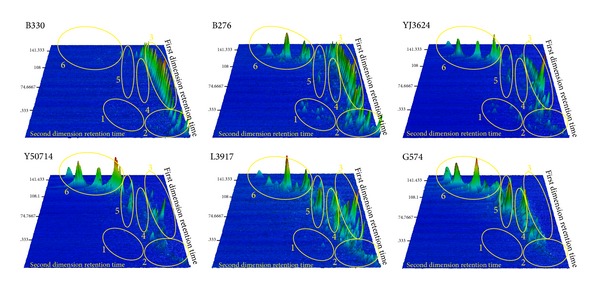
Main compounds of six oil samples in GC × GC/TOFMS TIC. 1: low molecular weight aromatic hydrocarbons; 2: light hydrocarbons; 3: n-alkanes and isoprenoids; 4: sesquiterpanes; 5 + 6: steranes and terpanes.

The total ion chromatograms (TIC) of oil B330 to oil G574 are shown in Figure 3, and the geochemical composition shows a significant difference. In order to describe this variation better, the overall composition of oil was divided into six zones (Figure 3).

During the degradation of oils (oil B330 to oil G574), compounds in Zone 3 were firstly consumed by microbes, and then their relative content decreased. The relative content of compounds in Zones 1 and 2 firstly increased and then reduced. Oil B276 had the highest relative content of compounds in Zones 1 and 2. The relative content of Zones 5 and 6 increased but the kind of compounds decreased. The relative content and the kind of compounds in Zone 4 all increased. GC/MS analysis indicated that the compounds in Zone 1 were low molecular weight aromatic hydrocarbons, and they were light hydrocarbons in Zone 2, n-alkanes and isoprenoids in Zone 3, sesquiterpenes in Zone 4, and steranes and terpanes in Zones 5 and 6, respectively. Additionally, peaks in GC × GC/TOFMS TIC of oil G574 have similar heights and colors. This demonstrates that the relative content of individual compounds in severely biodegraded oil (oil G574) is similar. In general, with the increase of degradation rank, the relative content of n-alkanes and isoprenoids decreased; light hydrocarbons and low molecular weight aromatic hydrocarbons increased first and then decreased; and some sesquiterpenes, steranes, and terpanes increased gradually, while the relative content of individual compounds in severely biodegraded oils tends to be similar.

#### 4.3.2. Compound Types

As shown in Figure 3, the compound types of severely biodegraded oil are different from that of normal oil. A brief classification of compounds in normal oil was presented in Section 4.3.1. The six compound types exhibit the variation in the degradation of oil. Firstly, the disappearance of types 1, 2, and 3 is the most significant feature. It is clearly shown by the absence of peak in Zones 1, 2, and 3 of oil G574. Secondly, many compounds in types 5 and 6 were degraded in severely biodegraded oil G574, such as C_27_ ~ C_29_-regular steranes, C_30_-hopane, and C_31_ ~ C_35_-homohopanes. The number of compound types can be represented by distribution area of peaks in the GC × GC/TOFMS TIC. The distribution area reduces gradually from oil B330 to oil G574, and the distribution area of the latter is only a quarter of that of the former. All the characteristics indicate that the compound types of biodegraded oil reduce significantly when compared with that of normal oil.

#### 4.3.3. Compound Polarity

Theorthogonal separation of GC × GC/TOFMS is achieved by the association of a nonpolar column with a polar column [28]. In this study, the first column is a nonpolar column and the second is a polar column. Therefore, the difference of the second dimension retention time represents the polarity difference of the compound [29].

Oil B330 has the “minimum” area in the second dimensional chromatographic plane because other compounds are covered by n-alkanes (Figure 3 B330), while oil B276 is more representative. The distribution area of oil B276 exceeds half of the second dimensional plane. In contrast, the peak area of oil G574 is much smaller, with only one-fifth of the second dimensional plane. The above difference indicates that the compounds in biodegraded oil have minor polarity difference.

In summary, compound types in biodegraded oils reduce significantly with the microbial degradation of n-alkanes, light hydrocarbons, isoprenoids, low molecular weight aromatic hydrocarbons, most steranes, and terpanes, residual compounds have minor difference in polarity, and the relative content of individual compounds tends to be similar. Thus the compounds of biodegraded oil are coeluted in the traditional GC analysis, resulting in the formation of the UCMs (“baseline hump”).

### 4.4. Identification of UCMs

GC × GC/TOFMS, liquid chromatography associated mass spectrometry, and other techniques have been applied to the study of UCMs in biodegraded oil and bitumen [6–9, 26, 28]. Mass spectra analysis showed that cyclic-alkanes may be an important component of UCMs. In this study, UCMs were further identified by the standard materials validation, structured chromatograms, and mass spectrums of target compounds.

#### 4.4.1. UCMs Classification of Oil G574

**Figure 4:**
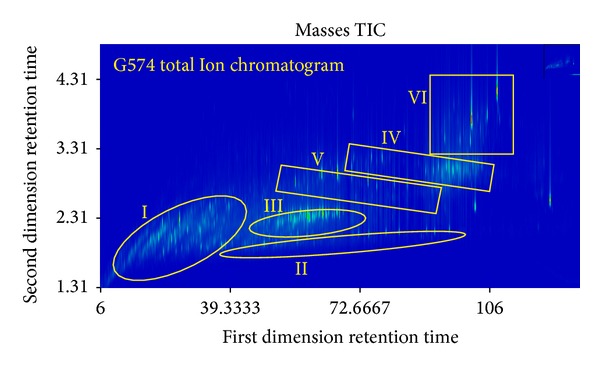
Classification of compounds in UCMs in severely biodegraded oil G574.

The UCMs in oil G574 were divided into six classes (I ~ *VI*⁡) according to the retention time and the spectrum structure (Figure 4). Under the collision of electrons with 70 ev in EI source, most molecules form fragment ions and part of molecules capture an electron to form molecular ions. Fragment ions can be used to infer molecular structure and molecular ions can determine the relative molecular mass. So the initial identification of compounds can be achieved [30]. Mass analyses of fragment ions showed that the characteristic ions of Class I are* M*/*Z*: 152, 166, 180, 194, 208, 222, and so forth, and they are* M*/*Z*: 126, 85 for Class II,* M*/*Z*: 123 for Class III,* M*/*Z*: 217 for Class IV, and* M*/*Z*: 191 for Classes V and VI, respectively. The composition of different classes was identified by different methods in the next section.

#### 4.4.2. Identification of UCMs Classes III to VI

**Figure 5:**
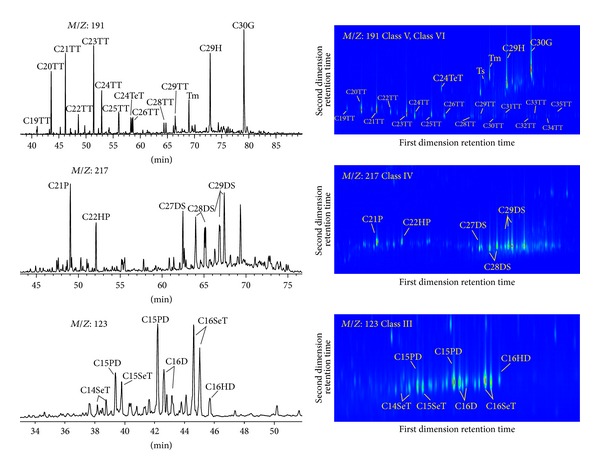
Qualitative diagram of compounds in classes III to VI. Left: GC/MS analysis; right: GC × GC/TOFMS analysis; TT: tricyclic terpane; TeT: tetracyclic terpane; H: hopane; G: gammacerane; P: pregnane; HP: homopregnane; DS: diasterane; SeT: sesquiterpane; PD: pentamethyl-naphthane; D: drimane; HD: homodrimane.

In order to determine the composition of UCMs for Classes III ~ *VI*⁡, characteristic ion chromatograms of GC/MS and GC × GC/TOFMS were compared (Figure 5). The comparison results indicated that Class III is of sesquiterpanes, Class IV is of pregnane, homopregnane, and diasteranes, Class V is of tricyclic terpanes, and Class VI is of norhopanes, gammacerane, and so forth. These compounds are common in normal oil and resistant to biodegradation, so they can remain in the severely biodegraded oil.

It should be pointed out that, for UCMs, Class III has bicyclic structure (sesquiterpanes), Class V has tricyclic structure (tricyclic terpanes), Class IV has tetracyclic structure (pregnane), and Class VI has pentacyclic structure (gammacerane). Cyclohexane is the basic framework of the cyclic structure. In addition to these compounds identified, a large number of small peaks appear within the range of UCMs for Classes III ~ *VI*⁡. Structural features of the two-dimensional spectra indicate that these peaks are the isomers of bicyclic to pentacyclic alkanes, and they are the major composition of UCMs for Classes III ~ *VI*⁡. UCMs of Classes III to VI are biomarkers, which are common in normal oils. So this part of UCMs is originated from the enrichment of hydrocarbons resistant to degradation in normal oils.

#### 4.4.3. Identification of UCMs Classes I and II

**Figure 6:**
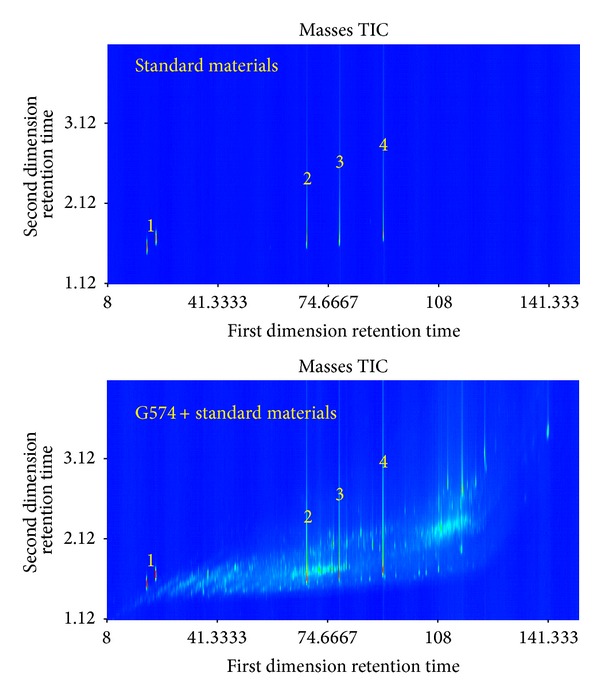
GC × GC/TOFMS TIC of standard materials and oil G574 spiked with standards. 1: naphthane (*cis*- and* trans*-); 2: dodecyl-cyclohexane; 3: tetradecyl-cyclohexane; 4: heptadecyl-cyclohexane.

Characteristic ions of UCMs Class I are* M*/*Z*: 138, 152, 166, 180, 194, 208, and 222 with features of isomer. The characteristic ions of UCMs of Class II are* M*/*Z*: 126 and 85. The first dimensional retention time of Class II increases gradually and the second dimensional retention time remains unchanged, which indicates that the compounds in Class II are isomers, too. According to the mass spectrums of target compounds and structured chromatograms, compounds in Class II may be the long chain alkyl-cyclohexanes. In order to validate the identification, standard materials naphthane (*cis*-,* trans*-), dodecyl-cyclohexane, tetradecyl-cyclohexane, and heptadecyl-cyclohexane were used. The GC × GC/TOFMS TIC of the standard materials and oil G574 spiked with standards are shown in Figure 6.

**Figure 7:**
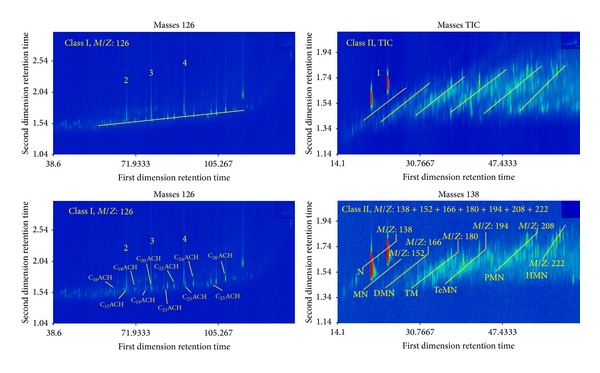
Composition of UCMs (classes I and II) identified by standard materials. 1, 2, 3, and 4 represent the same standards with Figure 6; ACH: alkyl-cyclohexane; N: naphthane; MN: methyl-naphthane; DMN: dimethyl-naphthane; TMN: trimethyl-naphthane; TeMN: tetramethyl-naphthane; PMN: pentamethyl-naphthane; HMN: hexamethyl-naphthane.

Additionally, the zoom of different unique ions is shown in Figure 7. It can be seen that naphthane (Standard 1) shows “roof-tile” effect with the compounds in Class II (Figure 7), which indicates that compounds in Class II are alkyl-naphthanes. The relative molecular mass of naphthane is 138 and the compounds with the characteristic ions of* M*/*Z*: 152, 166, 180, 194, 208, and 222 are methyl-, dimethyl-, trimethyl-, tetramethyl-, pentamethyl-, and hexamethyl-naphthane, respectively. Dodecyl-cyclohexane, tetradecyl-cyclohexane, and heptadecyl-cyclohexane (standards 2, 3, and 4) have almost the same first dimensional retention time and unique ions (*M*/*Z*: 126 and 85) as the compounds in Class I (Figure 7). It indicates that the compounds in Class I are long chain alkyl-cyclohexane. The substituents of long chain alkyl-cyclohexane were determined as C_10_ ~ C_20_ according to retention times of standard compounds 2, 3, and 4. In summary, Classes I and II were composed of alkyl-cyclohexanes, alkyl-naphthanes, and their isomers. The compounds in the two classes have complete series and are unusual in normal oil. Speculatively, UCMs classes I and II may come from the newly formed compounds during the microbial transformation of oil.

Based on the above analysis, the following conclusions can be drawn. Firstly, complex UCMs can be divided into six classes (Figure 4). UCMs are composed mainly of cyclic- to pentacyclic-alkanes, and cyclohexane is the basic framework of these cyclic alkanes. Secondly, the compounds in UCMs are alkyl-cyclohexanes, alkyl-naphthanes, sesquiterpenes, steranes, and terpanes that are resistant to degradation and their isomers. Thirdly, UCMs consist of both the existing hydrocarbons in crude oil and the newly formed compounds during the microbial transformation of oil.

## 5. Conclusions

Biodegraded oils B276 to G574 are typical lacustrine oils. The source rocks of the oils are the black mudstones in the Eh_3_^2^ of Biyang depression, and they are all low mature oils. The original compositions of this set of oils are comparable. The degradation ranks of oil B276 to oil G574 increase gradually.

With the degradation degree increasing (oil B330 to oil G574), n-alkanes, light hydrocarbons, isoprenoids, low molecular weight aromatic hydrocarbons, steranes, and terpanes are degraded in the relative order by microorganisms. The compound types of biodegraded oil reduce significantly, the compounds in different types have minor difference of polarity, and the relative content of individual compounds is similar. These changes result in the coelution of biodegraded oil in traditional GC analysis and form the raised “baseline hump” named as UCMs.

Standard compounds and mass spectrums analyses showed that cyclic alkanes with one to five rings (cyclohexane as basic framework) are major components of UCMs. UCMs can be divided into six classes (I ~ *VI*⁡). Classes I and II were composed of alkyl-cyclohexanes, alkyl-naphthanes, and their isomers. Classes III ~ *VI*⁡ were composed of sesquiterpenoids, tricyclic terpanes, low molecular weight steranes, diasteranes, norhopanes, and their isomers. Different classes of UCMs may have different sources. The former (I and II) are originated from the enrichment of hydrocarbons resistant to degradation in the oil and the latter (III ~ *VI*⁡) may come from the newly formed compounds during the microbial transformation of oil.

## Figures and Tables

**Table 1:** Reservoirs, depths, and group compositions of oil B330 to oil G574.

Sample	Reservoir	Depth (m)	Saturates (%)	Aromatics (%)	Resin (%)	Asphaltenes (%)
B330	Eh_3_^2^	1750.0	76.10	9.61	10.86	3.43
B276	Eh_3_^2^	1083.0	26.82	11.22	54.63	7.31
YJ3624	Eh_3_^2^	857.0	37.65	25.46	33.48	3.40
Y50714	Eh_3_^2^	753.0	25.98	19.22	49.35	5.45
L3917	Eh_3_^2^	282.0	43.10	16.70	36.80	3.39
G574	Eh_3_^2^	263.0	28.42	16.05	46.85	8.68

**Table 2:** Main geochemical parameters of oils from the northern slope.

Oil sample	Y3515	Y50714	YJ3624	B276	G412
C_27_/C_27_~C_29_-steranes	0.32	0.32	0.30	0.27	0.31
C_28_/C_27_~C_29_-steranes	0.33	0.33	0.34	0.33	0.31
C_19_/C_23_-tricyclic terpanes	0.05	0.03	0.03	0.12	0.07
C_19_~C_22_/C_23_~C_26_-tricyclic terpanes	0.62	0.50	0.50	1.17	0.74
C_26_-tricyclic/C_24_-tetracyclic terpane	2.24	2.32	2.38	1.72	2.51
C_21_~C_22_-pregnanes/(steranes + diasteranes)	0.02	0.02	0.01	0.03	0.03
C_19_~C_29_-tricyclic terpanes/*M*/*Z*:191	0.29	0.25	0.23	0.32	0.30
C_19_~C_29_-tricyclic terpanes/C_30_-hopane	1.30	1.12	0.97	1.49	1.33
C_27_~C_29_-diasteranes/steranes	0.05	0.05	0.05	0.06	0.06
T_s_/(T_s_ + T_m_)	0.15	0.15	0.15	0.10	0.14
C_31_~C_35_-homohopanes/C_30_-hopane	0.58	0.61	0.60	0.61	0.54

## References

[1] Hunt JM (1996). *Petroleum Geochemistry and Geology*.

[2] Head IM, Jones DM, Larter SR (2003). Biological activity in the deep subsurface and the origin of heavy oil. *Nature*.

[3] Peters KE, Moldowan JM (1993). *The Biomarker Guide: Interpreting Molecular Fossils in Petroleum and Ancient Sediments*.

[4] He SH (2010). *Oil and Gas Geology*.

[5] Gough MA, Rowland SJ (1990). Characterization of unresolved complex mixtures of hydrocarbons in petroleum. *Nature*.

[6] Ventura GT, Kenig F, Reddy CM (2008). Analysis of unresolved complex mixtures of hydrocarbons extracted from Late Archean sediments by comprehensive two-dimensional gas chromatography (GC×GC). *Organic Geochemistry*.

[7] Melbye AG, Brakstad OG, Hokstad JN (2009). Chemical and toxicological characterization of an unresolved complex mixture-rich biodegraded crude oil. *Environmental Toxicology and Chemistry*.

[8] Tran TC, Logan GA, Grosjean E, Ryan D, Marriott PJ (2010). Use of comprehensive two-dimensional gas chromatography/time-of-flight mass spectrometry for the characterization of biodegradation and unresolved complex mixtures in petroleum. *Geochimica et Cosmochimica Acta*.

[9] Wang HT, Zhang SC, Weng N (2013). Insight of unresolved complex mixtures of saturated hydrocarbons in heavy oil via GC×GC-TOFMS analysis. *Science China Chemistry*.

[10] Chen L, Wang H, Han J (2006). Sequence stratigraphy and stratum-lithology trap prediction of the Eh^3^ upper member of Hetaoyuan Formation in south Xia'ermen Oilfield, Biyang Sag. *Petroleum Exploration and Development*.

[11] Li HH (2008). Study of sequence stratigraphy and subtle reservoirs in the Biyang Sag of the Nanxiang Basin. * Petroleum Geology & Experiment*.

[12] Zhang J-G, Yao G-Q, Chen Y-B, Fan Z-H, Lin S-Q, Yang Y-L (2011). Sub-lacustrine fan of Chengdian and zircon U-Pb ages and constraint on its provenance in Biyang depression, Nanxiang Basin, China. *Journal of China University of Geosciences*.

[13] Dong T, He SH, Lin SQ (2013). Organic geochemical characteristics and thermal evolution maturity history modeling of source rocks in Eocene Hetaoyuan Formation of Biyang Sag, Nanxiang Basin. *Petroleum Geology & Experiment*.

[14] Luo JQ (2008). Geochemical characteristics and oil-source correlation of the immature to low-mature oil in the Biyang depression. *Geological Science and Technology Information*.

[15] Li SF, Hu SZ, He SH (2010). Oil-source correlation for biodegraded oils in the north slope of the Biyang Depression. *Acta Petrolei Sinica*.

[16] Hu SZ, Li SF, He SH (2010). Geochemical characteristics and significance of heavy oils from the west area of Biyang Sag, Nanxiang Basin. *Petroleum Geology & Experiment*.

[17] Seifert WK, Moldowan JM (1986). Use of biological markers in petroleum exploration. *Methods in Geochemistry and Geophysics*.

[18] Radke M (1988). Application of aromatic compounds as maturity indicators in source rocks and crude oils. *Marine and Petroleum Geology*.

[19] Wenger LM, Davis CL, Isaksen GH (2002). Multiple controls on petroleum biodegradation and impact on oil quality. *SPE Reservoir Evaluation & Engineering*.

[20] Peters KE, Walters CC, Moldowan JM (2005). *The Biomarker Guide: Biomarkers and Isotopes in Petroleum Exploration and Earth History*.

[21] Wei Z, Moldowan JM, Peters KE, Wang Y, Xiang W (2007). The abundance and distribution of diamondoids in biodegraded oils from the San Joaquin Valley: implications for biodegradation of diamondoids in petroleum reservoirs. *Organic Geochemistry*.

[22] Huang HP, Yang J, Larter SR (2003). Biodegradation effect on distributions of multiple methylated naphthalenes in reservoir extracts. *Earth Science—Journal of China University of Geosciences*.

[23] Bao JP, Zhu CS (2010). The effects of biodegradation on biomarker maturity indicators in sequentially biodegraded oils from Liaohe Basin, China. *Science in China D: Earth Sciences*.

[24] Ni CH, Bao JP, Yi G (2008). Study of biodegradation effect on aromatic biomarker parameters. *Petroleum Geology & Experiment*.

[25] Mao D, Weghe HVD, Lookman R, Vanermen G, Brucker ND, Diels L (2009). Resolving the unresolved complex mixture in motor oils using high-performance liquid chromatography followed by comprehensive two-dimensional gas chromatography. *Fuel*.

[26] van Mispelaar VG, Smilde AK, de Noord OE, Blomberg J, Schoenmakers PJ (2005). Classification of highly similar crude oils using data sets from comprehensive two-dimensional gas chromatography and multivariate techniques. *Journal of Chromatography A*.

[27] Sutton PA, Lewis CA, Rowland SJ (2005). Isolation of individual hydrocarbons from the unresolved complex hydrocarbon mixture of a biodegraded crude oil using preparative capillary gas chromatography. *Organic Geochemistry*.

[28] Adahchour M, Beens J, Vreuls RJ, Batenburg AM, Brinkman UAT (2004). Comprehensive two-dimensional gas chromatography of complex samples by using a “reversed-type” column combination: application to food analysis. *Journal of Chromatography A*.

[29] Tran TC, Logan GA, Grosjean E, Harynuk J, Ryan D, Marriott P (2006). Comparison of column phase configurations for comprehensive two dimensional gas chromatographic analysis of crude oil and bitumen. *Organic Geochemistry*.

[30] Watson JT, Sparkman OD (2007). *Introduction to Mass Spectrometry: Instrumentation, Applications, and Strategies for Data Interpretation*.

